# Host Cyanobacteria Killing by Novel Lytic Cyanophage YongM: A Protein Profiling Analysis

**DOI:** 10.3390/microorganisms10020257

**Published:** 2022-01-24

**Authors:** Shanshan Zhang, Baohua Zhao, Jing Li, Xiaofei Song, Yigang Tong, Wenlin An

**Affiliations:** 1 College of Life Science, Hebei Normal University, Shijiazhuang 050024, China; zhangshanshanhbtu@163.com (S.Z.); zhaobaohua@hebtu.edu.cn (B.Z.); 2 College of Life Science and Technology, Beijing University of Chemical Technology, Beijing 100029, China; frostli@163.com (J.L.); 13261297988@163.com (X.S.); tongyigang@mail.buct.edu.cn (Y.T.); 3 Department of Scientific Research Management, National Vaccine and Serum Institute, Beijing 100176, China

**Keywords:** cyanophage, *Nostoc* sp., label-free quantitative proteomics, photosynthesis, substance metabolism, energy metabolism

## Abstract

Cyanobacteria are autotrophic prokaryotes that can proliferate robustly in eutrophic waters through photosynthesis. This can lead to outbreaks of lake “water blooms”, which result in water quality reduction and environmental pollution that seriously affect fisheries and aquaculture. The use of cyanophages to control the growth of cyanobacteria is an important strategy to tackle annual cyanobacterial blooms. YongM is a novel lytic cyanophage with a broad host spectrum and high efficiency in killing its host, cyanobacteria FACHB-596. However, changes in cyanophage protein profile during infestation and killing of the host remains unknown. To characterize the proteins and its regulation networks involved in the killing of host cyanobacteria by YongM and evaluate whether this strain YongM could be used as a chassis for further engineering to be a powerful tool in dealing with cyanobacterial blooms, we herein applied 4D label-free high-throughput quantitative proteomics to analyze differentially expressed proteins (DEPs) involved in cyanobacteria host response infected 1 and 8 h with YongM cyanophage. Metabolic pathways, such as photosynthesis, photosynthesis-antennal protein, oxidative phosphorylation, ribosome, carbon fixation, and glycolysis/glycol-isomerization were significantly altered in the infested host, whereas DEPs were associated with the metabolic processes of photosynthesis, precursor metabolites, energy production, and organic nitrogen compounds. Among these DEPs, key proteins involved in YongM-host interaction may be photosystem I P700 chlorophyll-a apolipoprotein, carbon dioxide concentration mechanism protein, cytochrome B, and some YongM infection lysis-related enzymes. Our results provide comprehensive information of protein profiles during the invasion and killing of host cyanobacteria by its cyanophage, which may shed light on future design and manipulation of artificial cyanophages against water blooms.

## 1. Introduction

Water eutrophication is a global water pollution problem that causes massive production of cyanobacteria [1,2]. The latter can lead to a cyanobacteria bloom, causing huge economic and environmental losses [3,4,5]. Cyanophages are a group of viruses that specifically infect prokaryotic cyanobacteria [6,7,8,9], playing a very important role in the regulation of the population and influencing evolution [10]. These viruses are biological control agents and have great potential for controlling harmful blooms of cyanobacteria [11]. However, most reported cyanophages have strong host specificity, a narrow cyanobacteria-killing spectrum, and a long lysis cycle [12,13]. For example, MA-LMM01, isolated from Lake Mikata, Japan [14], could only infect a toxic strain of *Microcystis aeruginosa* and had a long incubation period [15,16]. The *Podoviridae* cyanophages Pf-WMP3 [17] and Pf-WMP4 [18] can infect the same host cyanobacteria, but with a narrow algaecidal spectrum. Infection experiments have shown that S-LBS1 takes about four days to induce cyanobacterial lysis, and only in strain TCC793 of *Synechococcus* sp. [19]. In addition, newly discovered cyanophages in the last 2–3 years, such as PA-SR01, only lysed *Pseudanabaena* sp. KCZY-C8 [20]. Mic1 was only infective to its host, *Microcystis wesenbergii* FACHB-1339 [21]. Cyanophages S-H68 and S-B68, isolated from Bohai Sea waters, had incubation periods as long as 41 and 49 h, respectively [22,23]. Cyanobacterial blooms are usually formed by mixed outbreaks of multiple cyanobacteria with a rapid reproduction rate [24,25]. Therefore, application of existing cyanophages to treat cyanobacterial blooms is limited [26,27].

Cyanophage infection of cyanobacteria involves multiple proteins, and conventional methods cannot systematically analyze their mechanisms of action in a high throughput fashion. The quantitative proteomics system is a powerful approach in investigating the pivotal proteins involved in the cyanophage’s infection of host cyanobacteria and deciphering the cyanobacteria-killing mechanism of cyanophages. Studies on *Prochlorococcus* sp. MED4 and its cyanophage P-SSP7 have shown that infection is followed by complex gene expression dynamics, but little is known about the initial cyanophage-host interactions [28]. The infection of the cyanophage S-SM1 with *polycoccus* WH8102 has been studied with an isotope-labeled proteomic approach to determine the uptake of extracellular nitrogen and its use in biosynthesis of viral and host proteins [29]. The effect on photosynthesis of the cyanophage P-TIM68 carrying PSI and PSII-related genes during infection of the *Prochlorococcus* host MIT9515 in tropical oceans showed that P-TIM68 may maintain or even manipulate host photosynthesis [30]. Genomics, proteomics, and transcriptomics have been used to trace Shine–Dalgarno sequence differences in *Synechocystis* sp., *Microcystis aeruginosa*, cyanophage, tobacco chloroplasts, and *Arabidopsis chloroplasts* [31]. However, off-label quantitative proteomics have not been used to investigate the mechanism of cyanophage infestation of host cyanobacteria. Also, further research is needed on the kinetics, phagocytosis pattern, and mechanism of interaction between the cyanophage and host [32].

The newly isolated *Myoviridae* cyanophage from water samples of Kunming Dianchi Lake, YongM, has efficient lysis characteristics and can rapidly infest and lyse its host *Nostoc* sp. FACHB-596 within 24 h. YongM was able to lyse 18 of 33 tested cyanobacteria strains, indicating a broad spectrum of lysis hosts. The lysis characteristics of YongM have been studied by exposure experiments and absorbance measurements. The color change of the host solution was not obvious 1 h after YongM infestation, but it turned yellow after 8 h, representing the lysis phase. This lysis efficiency is more rapid and efficient than any strain reported so far and it can be potentially applied to control cyanobacterial blooms. Therefore, the host cyanobacteria 1 and 8 h after YongM infestation were used for quantitative proteomic comparison. DEPs in the host were screened and the expression characteristics were bioinformatically analyzed. GO analysis revealed that after infection, most of the DEPs were associated with photosynthesis, light response, precursor metabolites and energy production, and metabolic processes of organic nitrogen compounds. KEGG pathway enrichment results indicated that metabolic pathways, such as photosynthesis, photosynthesis-antenna protein, oxidative phosphorylation, ribosome, carbon fixation, and glycolysis/glycoisomerization were significantly altered in the infested host cyanobacteria. In addition, there were several key, differentially expressed proteins, such as photosystem I P700 chlorophyll-a apolipoprotein, carbon dioxide concentration mechanism protein, and cytochrome B. These may serve as hotspots for the study of the YongM-host interaction mechanism. They can also provide genetic resources and a theoretical basis for future cyanophage transformation to produce efficient and broad-spectrum cyanophages, and to understand their interaction with the hosts.

## 2. Materials and Methods

### 2.1. Materials

The strain of host cyanobacteria FACHB-596 was obtained from a freshwater cyanobacteria culture bank at the Institute of Hydrobiology, Wuhan Academy of Sciences, China. Cyanophage YongM was isolated from water samples of the Dianchi Lake in Kunming, Yunnan. It was preserved in the Center for General Microbiology of Microbial Culture Collection Management Committee, Conservation Number: CGMCC No.18383.

BG-11 medium was purchased from Qingdao Hope Bio-Technology Co., Ltd, Qingdao, China. (product number: HB8793). Its ingredients include sodium nitrate, potassium hydrogen phosphate trihydrate, magnesium sulfate heptahydrate, calcium chloride dihydrate, citric acid, ammonium ferric citrate, EDTA, sodium carbonate, boric acid, manganese chloride monohydrate, zinc sulfate heptahydrate, copper sulfate pentahydrate, sodium molybdate dihydrate, and cobalt nitrate hexahydrate. A GXZ intelligent light incubator was purchased from Ningbo Jiangnan Instrument Factory, Ningbo, China (model number: GXZ-280B).

### 2.2. Separation, Purification, and Suspension Preparation

Samples of surface water were collected from Yunnan Dianchi (latitude: 24.964616, longitude: 102.666303) and centrifuged at 12,000× *g* for 20 min at 4 °C. The supernatant was filtered through 0.45 μm and 0.22 μm pore-sized nitrocellulose membranes (Merck Millipore Ltd., Shanghai, China). The filtrate was added to a volume ten times larger of FACHB-596 cyanobacteria in logarithmic growth solution. The solution was mixed and incubated until yellowing was evident. The procedure was repeated three times. The produced lysate was then serially diluted ten-fold with BG-11 medium and used in double-layer agar plate spread experiments. After the growth of phagocytic spots were observed, several individual spots were dug up and suspended in 5 mL of FACHB-596 growth suspension in logarithmic phase. The above phagocytic spot experiment was repeated after yellowing, until phagocytic spots of uniform shape and size were formed on the plate. The purified lysate was centrifuged at 5000× *g* for 10 min at 4 °C, producing a suspension of purified YongM in the supernatant.

### 2.3. Genome Enrichment and Extraction

High titer cyanophage-cyanobacteria culture lysate (30 mL) was centrifuged at 6000× *g* for 20 min, and the supernatant was sequentially filtered through 0.45 μm and 0.22 μm pore size nitrocellulose membranes. The filtrate was centrifuged in a density gradient at 4 °C for 1 h at 35,000× *g* using 20% (*w*/*v*) and 40% (*w*/*v*) sucrose solution freshly prepared within a week. After discarding the supernatant, the precipitate was resuspended with 200 µL of PBS (0.01 M, PH7.4) and the cyanophage genome was extracted using the Roche High Pure Viral Nucleic Acid Kit (Roche, Basel, Switzerland).

### 2.4. Infection and Sample Collection

The host culture, in logarithmic phase, was inoculated with a YongM suspension at an optimal MOI = 0.1 (experimental group T) whereas another sample was inoculated only with BG-11 medium (control group C). Each group was placed in a light incubator at 25 °C with a light intensity of 2000 lx and exposed to 12 h light-dark cycles. After 1 h and 8 h of inoculation, the cultures in both groups were gently shaken and a sample (3 mL) was taken immediately from each group. These samples were centrifuged at 6000× *g* for 15 min at 4 °C. The sediments were washed twice with PBS and stored at −80 °C for protein extraction and proteome sequencing.

### 2.5. Protein Extraction and Digestion

For extraction, lysis buffer (500 μL, 8 mM urea, 30 mM HEPES, 1 mM PMSF, 2 mM EDTA and 10 mM DTT) was added to alga cells, followed by a 5 min ultrasonic ice bath treatment (pulse on 2 s, pulse off 3 s, power 230 W). The supernatant was centrifuged at 2000× *g* for 30 min. DTT was added until the final concentration was 10 mM. After the samples were incubated at 56 °C for 1 h, IAM was quickly added to a final concentration of 55 mM and the solution was incubated in the dark for 1 h. The protein concentration was quantified by the Bradford method.

For digestion, 40 μg protein from each sample was centrifuged in ultrafiltration tubes with 3 K MCWO at 14,000× *g* (4 °C, 40 min) and the supernatant was discarded. The pellet was solubilized with 200 μL of 50 mM NH_4_HCO_3_ and was centrifuged at 4 °C for 40 min at 14,000× *g*, discarding the supernatant. The process was repeated twice. Trypsin (1 μg/μL) was added to the samples at a protein substrate to enzyme ratio of 30. The samples were incubated in water at 37 °C for 24 h. The digested samples were lyophilized and solubilized with 25 mM NH_4_HCO_3_ (30 μL per tube).

### 2.6. Proteome Sequencing

Desalted peptide mixtures were loaded onto a Acclaim PePmap C18-reversed phase column (75 μm × 2 cm, 3 μm, 100 Ǻ thermo scientific) and separated with a reversed phase C18 column (75 μm × 10 cm, 5 μm, 300 Ǻ, Agela Technologies, Tianjin, China) mounted on a Dionex ultimate 3000 nano LC system. Peptides were eluted using a gradient of 5–80% (*v*/*v*) acetonitrile in 0.1% formic acid over 45min at a flow rate of 300 nL min^−1^ combined with a Q Exactive mass spectrometer (Thermo Fisher Scientific, Waltham, MA, USA). The eluates were directly entered using Q—Exactive MS (Thermo Fisher Scientific, Waltham, MA, USA), setting in positive ion mode and data-dependent manner with full MS scan from 350–2000 *m*/*z*, full scan resolution at 70,000, MS/MS scan resolution at 17500.MS/MS, scan with minimum signal threshold 1 × 10^5^, isolation width at 2 Da. To evaluate the performance of this mass spectrometry on the Label-free samples, two dd-MS2 acquisition modes was used, AGC target 1 × 10^5^, normalized collision energy (NCE) was systemically examined 30, stepped 27%.

### 2.7. Data Analysis

The free software MaxQuant (version 1.6.0.1) (developed by Jürgen Cox & Matthias Mann in Max-Planck Institute for Biochemistry, Martinsried, Germany) was used for mass spectrometry data acquisition and quantitative processing. MSstats ANOVA in R software was used to assess the significance of differences. DEPs were defined as those with fold change (FC) greater than or equal to 1.2, or less than 0.833, between experimental and control groups by *t*-test *p* < 0.05. The PCA analysis, volcanic maps, heat map of DEPs clustering, and associated variance data were analyzed by GraphPad Prism (8.0.2) and R Package (V.2.0.3). To determine the biological role of DEPs, we used Gene Ontology (GO) analysis with the Gene Ontology database and the annotation and classification of proteins according to the three aspects of GO analysis, namely biological processes, cellular components, and molecular functions. The Kyoto Encyclopedia of Genes and Genomes database (KEGG) was used to identify DEP-related metabolic and signal transduction pathways.

## 3. Results and Analysis

### 3.1. Effect of YongM Infection on Host Growth

**Figure 1:**
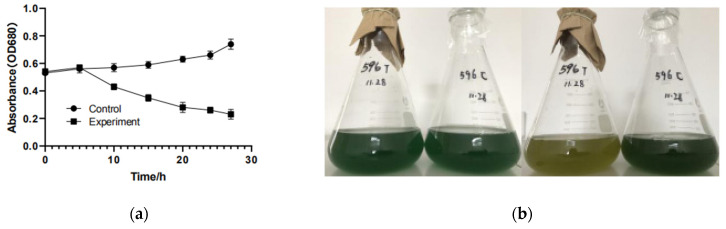
Growth of host cyanobacteria FACHB-596 after cyanophage YongM infection. (**a**) Host cyanobacteria FACHB-596 absorbance versus infection time by YongM; (**b**) Phenotypic changes of YongM-infected host cyanobacteria FACHB-596 cells after 1 h (**left**) and 8 h (**right**).

YongM was added to log-phase host cyanobacteria FACHB-596 cells and was incubated under a circadian rhythm (alternate 12 h dark and light periods). The color change of the cyanobacteria solution is not obvious after 1 h, where YongM is still in the latent phase, but the solution turned yellow after 8 h (Figure 1), indicating that the phagosome was in lysis phase. The susceptibility of 33 cyanobacterial strains to YongM is shown in Table 1.

### 3.2. Altered FACHB-596 Protein Expression Profile Caused by YongM Infection

**Figure 2:**
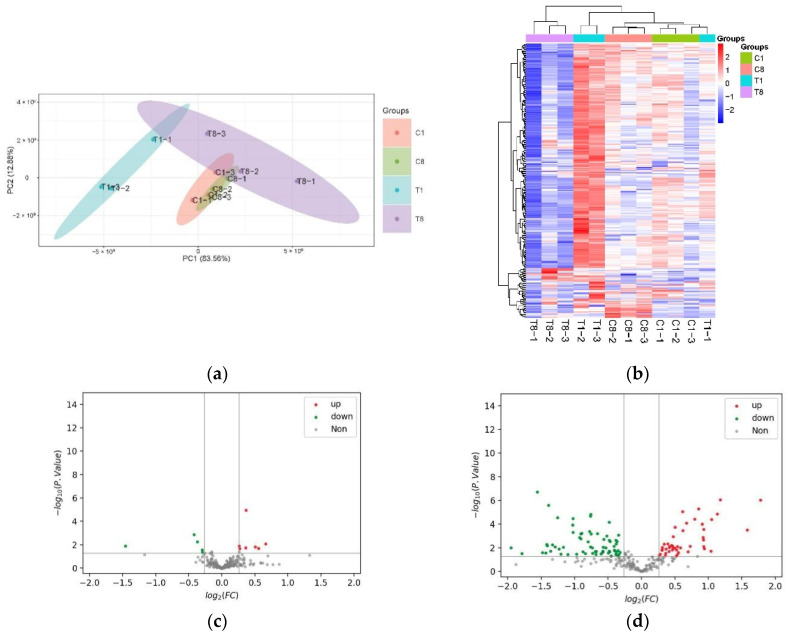
Changes in protein expression in host cyanobacteria caused by YongM infection. (**a**) PCA of DEPs; (**b**) heat map; (**c**,**d**) volcanic maps of DEPS after 1 h infection (**c**) and 8 h infection (**d**).

Logarithmic stage host cyanobacteria FACHB-596 infested with YongM after 1 h and 8 h were used as experimental groups (T1 and T8, respectively), whereas cells not exposed to the cyanophage were used as control (C1 and C8, respectively). Proteomic and bioinformatic analyses showed up to 573 proteins differentially expressed after cyanophage infection. Principal component analysis (PCA) of these DEPs (Figure 2a) showed a small separation between the samples in the same group, but a large separation for samples belonging to different groups. This indicates that the experiments were reproducible, and the data was reliable. This also suggests that there are protein expression changes between experimental and control groups.

To visualize the changes in the FACHB-596 cyanobacteria cells altered proteome caused by YongM infection, DEPs were clustered and heatmaps were drawn (Figure 2b). A total of 12 DEPs (5 down-regulated and 7 up-regulated) were significantly altered after 1 h of infection compared to the control (Figure 2c). The most down-regulated protein was the 50S ribosomal protein L23 (A0A1Z4KLJ5_ANAVA), whereas the most up-regulated protein was the ATP-dependent zinc metalloproteinase FtsH (Q8YXF2_NOSS1). After 8 h, 112 DEPs (67 down-regulated and 45 up-regulated) were altered (Figure 2d). The most down-regulated protein being peroxidase 2 family protein/glutaminase (Q8YWR3_NOSS1) and the most up-regulated protein being photosystem I P700 chlorophyll-a apolipoprotein A1 (A0A1Z4M5R2_MICDP).

### 3.3. Functional Analysis of DEPs

The number of DEPs individually expressed during 1 h and 8 h after infection in the YongM-infected and uninfected host cells were 127, 74, 74 and 43, respectively. The top 3 statistics of DEPs (Table 2) show that after 1 h of infection, only the membrane-bound, ATP-dependent zinc metalloproteinase FtsH, generally conserved in prokaryotes, was significantly up-regulated. This protein degrades proteins with low thermodynamic stability and lacks unfolding enzyme activity. This suggests that it may be part of a prokaryotic self-protection mechanism to check whether proteins are folded correctly. Ribosomal protein S14, one of the major constituent proteins of small subunit 30 S, is significantly up-regulated in bacteria, cyanobacteria, and plants, indicating enhanced translational activity and high protein synthesis. An additional All4042 protein of unknown function was also significantly up-regulated. The significantly down-regulated outer membrane efflux protein (Alr2887 protein) forms a trimeric channel that can export a variety of substrates in Gram-negative bacteria. After 8 h of infection, photosystem I P700 chlorophyll-a apolipoprotein A1 was significantly up-regulated. This is a membrane protein complex that uses light energy to mediate the transfer of electrons from plastid proteins to ferric oxide reducing proteins and is primarily involved in cyanobacterial photosynthesis. Carbon dioxide concentration mechanism protein is present in a number of autotrophic and non-autotrophic multiple polyhedral shell proteins (CcmK), therefore it may be related to CO_2_ utilization. A significantly down-regulated protein was the late competence development protein (A0A1Z4IAM0) that produces an adaptive response to external stimuli and is required for cellular uptake of exogenous DNA from the environment leading to transforming capacity. The latter is prevalent in bacteria and may be involved in gene transfer. The significantly down-regulated photosystem I reaction center subunit IV, present on the matrix side of the cystoid membrane, can form complexes with ferricoxigenin and ferricoxigenin oxidoreductase at the photosystem I reaction center and is associated with cyanobacterial photosynthesis. The phycocyanin β subunit, a phycobilisome protein, collects light energy through water-soluble complexes of the phycobilisome, which are attached to the outer surface of the cystoid membrane and can transfer absorbed energy to the photosynthetic reaction center with >95% efficiency.

In summary, after 8 h of infection, host cells showed a weakened defense against exogenous nucleic acid. Expression of argininosuccinate synthase was reduced, resulting in the inhibition of the penultimate step in arginine biosynthesis. This makes it impossible to form argininosuccinate, AMP, and pyrophosphate from citrulline and aspartate.

### 3.4. GO Analysis of DEPs

**Figure 3:**
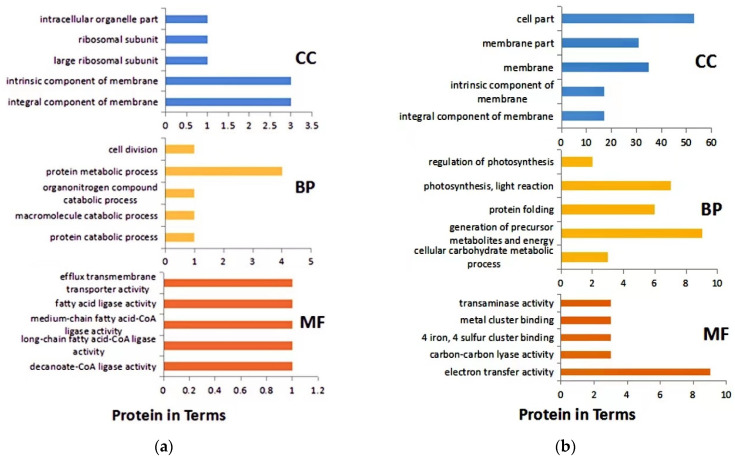
GO analysis of DEPs. (**a**) after 1 h infection; (**b**) after 8 h infection. BP: Biological process; CC: Cell composition; MF: Molecular function.

GO analysis revealed that the main biological process of DEPs after 1 h of infection was protein metabolism. Cellular components were mainly intrinsic to the membrane, and molecular functions were mainly long- and medium-chain fatty acid-coenzyme ligase activity and fatty acid ligase activity (Figure 3a). After 8 h of infection, the main biological processes of DEPs were precursor metabolites and energy production, photosynthesis, and light reaction. The cellular components were mainly cells and their cell membrane parts. The molecular functions were mainly related to electron transfer activity (Figure 3b). In addition, DEPs were also involved in carbon fixation in photosynthesis, amino acid synthesis, and biosynthesis of antibiotics.

### 3.5. KEGG Pathway Analysis of DEPs

**Figure 4:**
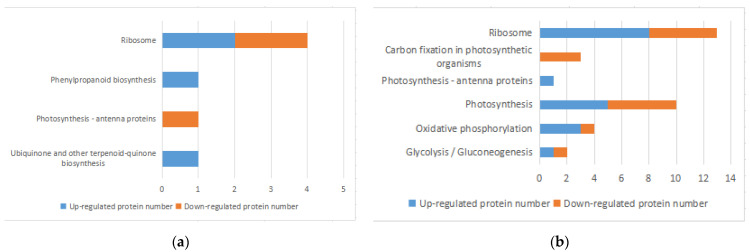
Enrichment of KEGG pathway in DEPs. (**a**,**b**) KEGG enrichment histogram of DEPs after 1 h infection (**a**) and after 8 h infection (**b**).

KEGG Pathway analysis is the analysis of information networks connecting known molecular interactions, such as metabolic pathways, complexes, and biochemical reactions. The KEGG Pathway was used to analyze DEPs through the MaxQuant Cloud Platform for Integrative Analysis of Genomic Data Collection. Four metabolic pathways, involving 7 DEPs, were significantly enriched 1 h after infections, and 4 of them showed elevated DEPs, accounting for 57.1% (Figure 4a). A total of 41 pathways involving 141 DEPs were enriched 8 h after infection, and six metabolic pathways were significantly enriched with DEPs common to all of them. For example, the photosynthesis-related protein (A0A5Q0GEQ2) was involved in both photosynthesis-related and oxidative phosphorylation metabolic pathways (Figure 4b). Six metabolic pathways were significantly enriched after 1 h and 8 h containing 39 DEPs (Table 3). Among the DEPs found in all pathways, 116 DEPs down-regulated (82.3%), suggesting that 8 h after infection most protein expression in the host cyanobacteria was inhibited, resulting in a significant decrease in metabolic activity and a weakening of the vital processes. Enriched pathways (Figure 4) included those directly related to cyanobacteria pigment synthesis and energy supply, such as ribosome metabolism, carbon fixation, oxidative phosphorylation, energy metabolism, photosynthesis, and secondary metabolites (e.g., phenylpropanoids and dicarboxylic acids).

#### 3.5.1. Analysis of Photosynthetic DEPs

The phycobilisomes consist of multiple photosynthetic light-harvesting proteins (i.e., photosynthesis-associated haptoglobin) attached to the outer surface of the cystoid membrane [33]. Cyanobacteria collects light energy through multiple light-harvesting proteins in this complex and transfers the absorbed energy to photosynthetic reaction centers with an efficiency greater than 95%. Among these light-harvesting proteins, phycocyanin is the core component of the water-soluble complex, receiving and transferring energy to the chlorophyll of the cystoid membrane [34]. When YongM infested the host for 1 h, the expression of phycocyanin in the host was reduced, the light-harvesting ability was weakened whereas photosynthesis was inhibited. In contrast, after 8 h YongM infiltration, we observed elevated phycocyanin-related rod-linked proteins, which contributed to light uptake and increased photosynthetic efficiency.

The PSII oxygen precipitation complex (OEC) consists of five subunits, PsbO, PsbP, PsbQ, PsbU, and PsbV [35], which can be photolyzed by water to provide protons for PSI. Its P680 reaction center contains chlorophyll a, which photolyzes water and produces ATP using a proton pump [36]. The P700 reaction center of PSI contains chlorophyll, which absorbs electrons and associated hydrogen delivered from PSII to reduce NADP+ to NADPH. Both ATP and NADPH are then used in light-independent dark reactions, culminating in the conversion of carbon dioxide to glucose and the release of oxygen as a byproduct. PSII is a multisubunit protein-pigment complex in which the core, chlorophyll, and β-carotene bind mainly to the haptoglobin CP43 (PsbC) and CP47 (PsbB), which transmit excitation energy to the reaction center proteins PsbA and PsbD, combining all redox-active cofactors involved in energy conversion [37,38]. After 8 h of infection, the subunits responsible for water photolysis, PsbO, PsbP, PsbQ, and PetE related to photosynthetic electron transport were all down regulated, whereas the photoresponsive phase was suppressed. The up-regulated proteins were Psb27, a highly conserved component of photosystem II consisting of four helices [39], PsaA, a membrane-integrated protein involved in constituting PSI [40] and the cytochrome complex PetA [41], a protein associated with senescence whose up-regulation may indicate that photosynthesis was almost stagnant, and cells were in a state of senescence.

In addition to the above pathway, all DEPs in the carbon fixation pathway were down-regulated after 8 h; phosphoglycerate kinase (PGK), present in all living organisms, catalyzes the interconversion of ATP and ADP and is highly conserved throughout evolution, converting 1,3-diphosphoglycerate to 3-phosphoglycerate in the second step of glycolysis and forming a molecule of ATP [42]. In most cells, this reaction is essential for ATP production in aerobic bacteria, fermentation in anaerobic bacteria, and carbon fixation in plants. Ribulose diphosphate carboxylase large subunit (RbcL) is the macromolecular subcomplex site of Rubsico, and the RbcX protein has been identified as possessing a chaperone-like protein function, as it contributes to the correct assembly of RbcL and RbcS subunits during Rubsico biosynthesis [43]. This metabolic pathway is complemented by down-regulated pyruvate phosphate dikinase, which catalyzes the reversible conversion of ATP to AMP, pyrophosphate, and phosphoenolpyruvate (PEP). These two enzymes are essential for carbon fixation in cyanobacteria cells, resulting in an inadequate energy supply mechanism during carbon fixation and blocked organic matter synthesis in the host cyanobacteria.

#### 3.5.2. Analysis of Energy Metabolism DEPs

After 8 h of infection, expression of ubiquinone oxidoreductase, an enzyme that catalyzes the transfer of electrons from NADH to ubiquinone and is associated with proton translocation across membranes, was reduced. This is the largest and most complex enzyme in the respiratory chain. In cyanobacteria, the three subunits NdhM, NdhN, and NdhO of the NDH-1 complex influence the electron flow around PSI by providing additional ATP for plastids and photosynthesis in cyanobacteria [44]. The NADH dehydrogenase I complex transfers electrons through FMN and Fe-sulfur centers to quinones in the respiratory and photosynthetic chains and couples redox reactions to proton transport, resulting in proton redox energy conserved as a proton gradient [45,46]. Also, among the upregulated expression proteins, alpha is transmembrane ATPases, which are membrane-bound enzyme complexes/ion transport proteins that use ATP hydrolysis to drive proton translocation across membranes. There are also transmembrane ATPases that work in reverse, using energy in the proton gradient and transmembrane ion flow across the ATPase proton channel to drive ATP synthesis. b is F-ATPase, also known as ATP synthase, a type of transmembrane ATPase that hydrolyzes ATP in bacteria to generate a proton gradient [47,48]. The above suggests that infection by YongM results in the restriction of proton transport during oxidative phosphorylation of the host.

In addition, two DEPs, one up-regulated and another down-regulated, were involved in the glycolytic and gluconeogenic metabolic pathways after 8 h of infection. The up-regulated protein is the 2-oxoacid dehydrogenase acyltransferase, which catalyzes the overall conversion of α-ketoacid to acyl-CoA and CO_2_ [49], whereas the down-regulated one is PGK, which is involved in the carbon fixation pathway that is also down-regulated. This protein could catalyze the interconversion of ATP and ADP. Thus, the infestation of YongM may prevent host glycolysis and gluconeogenesis pathways from proceeding normally by regulating the overall conversion of α-keto acid to acyl-CoA and carbon dioxide and the expression of catalytic enzymes that convert ATP and ADP.

#### 3.5.3. Analysis of Translation Function DEPs

Ribosomes are organelles present in all organisms that use mRNA as a template for the targeted synthesis of genetic information carried by genes. About 1/3 of the ribosome mass consists of proteins that are named small subunit proteins (S1 to S31) and large subunit proteins (L1 to L44), based on their ribosomal subunit size. Many ribosomal proteins, especially those of the large subunit, have long finger-like protrusions that extend into the rRNA core and stabilize their structure. In the large subunits, about 1/3 of the 23S rRNA nucleotides are at least in Van der Waals contact with the protein, while L22 interacts with all six structural domains of the 23S rRNA. The proteins S4 and S7 of the starting 16S rRNA assembly are located at the junctions of five and four RNA helices, respectively, and can be used to organize and stabilize the tertiary structure of the rRNA [50,51].

After 1 h of exposure, we observed a decrease in protein expression of ribosomal large subunit proteins L25 and L2, which are known to bind 23S rRNA and have peptidyl transferase activity in *E. coli*. Among the proteins with elevated expression, S14 is one of the proteins from the small subunit of the ribosome. This protein is required for the assembly of 30S particles in *E. coli* and may also be responsible for determining the conformation of 16S rRNA at the A site [52]. Cyanobacteria are Gram-negative bacteria, and the function of its ribosomal proteins is similar to that of *E. coli*. After 8 h of infection, the number of proteins with elevated expression increased significantly, e.g., L1, the largest protein from the ribosomal subunit, with RNA binding site highly conserved and RNA chaperone activity. Compared with 1 h, differentially expressed ribosomal proteins were significantly increased after 8 h. Five large subunit proteins associated with ribosomal rRNA core stability were up-regulated (L29, L13, L1, L35, L21) which facilitate the stable translation of mRNA and sustained protein synthesis. In addition, the expression of the S5 protein, which is closely related to the function of 30S small subunit assembly and can reduce the rate of translation errors, was also up regulated. We speculate that this is consistent with the high vital activity of the cyanophage after 8 h, when they are in the stage of cleavage.

### 3.6. Structural and Functional Proteins of YongM

**Figure 5:**
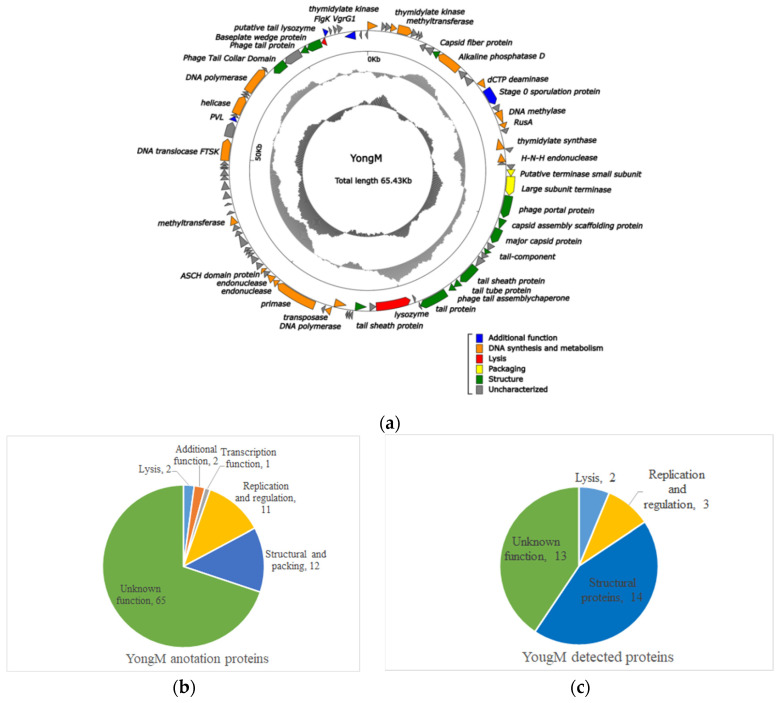
Comparison of predicted ORFs of YongM with proteins identified by proteomics. (**a**) Genome map of YongM; (**b**) heat map of DEPs due to cyanophage YongM infestation of host cyanobacteria FACHB-596 cells; (**c**) protein function/metabolic pathway and proportion of YongM detected proteins.

Amplification, concentration, and mass spectrometry (LC-MS/MS) analysis of YongM were performed to further validate the results obtained. By delineating and comparing the functional modules of the predicted Open Reading Frames (ORFs) and the actual identified proteins by mass spectrometry (Figure 5b,c), we found that 32 proteins (33.34% of the predicted ORFs) out of 93 predicted proteins (Figure 5a) in the YongM genome could be identified by mass spectrometry proteomics. These included 14 structural proteins, 5 functional proteins, and 13 unknown functional proteins (Table 4), most of which were encoded by the identified structural genes. The molecular weights of the identified proteins were very close to the predicted values. In addition, we identified two lysozymes (Anabaena phage Elbi) and a single lysozyme (Nostoc phage N1) by protein profiling, encoded by the predicted ORFs at position 41 and 86 of the YongM genome, with sizes of 99 kDa and 12.65 kDa, respectively. Four genes were related to DNA anabolism: thymidylate kinase, DNA polymerase, nucleic acid endonuclease, and alkaline phosphatase, which may contribute to the rapid infection and proliferation of YongM [53]. Therefore, we speculate that the lysis efficiency of YongM is inextricably linked to its own dual lytic enzymes and DNA synthesis-related proteins. In addition, the ability of YongM to lyse its host within 12 h and its relatively wide host range may also be attributed to its multiple restriction endonucleases and methylesterases. YongM may serve as a model for future studies on the interaction between these enzymes and freshwater cyanobacteria.

## 4. Discussion and Conclusions

Since the discovery and isolation of cyanophages, their biological functions and ecological importance in aquatic ecosystems have received great attention, especially in the control of harmful cyanobacterial blooms. Indeed, cyanophages have become important focal points in environmental science and virology. In recent years, molecular biology methods have been used to study the genetic diversity and evolutionary history of cyanophages [54,55]. For example, transcriptomic and metabolomic analyses can be performed on lytic cyanophage-infected host cells. However, gene expression can also be regulated at the post-translational level, therefore proteomic analyses are essential to fully understand the cellular response to specific conditions and to track the fate of each protein [56]. However, the systems involved in the regulation of protein expression levels are very complex, producing a very dynamic proteome. Thus, technical limitations have left most of the viral proteome unresolved, except in a few cases of classic examples [57]. Recent improvements in instrumentation, and the development of highly sensitive analytical methods, have facilitated advances in viral proteomics, but most studies are still focused on the biology of eukaryotic viruses.

**Figure 6:**
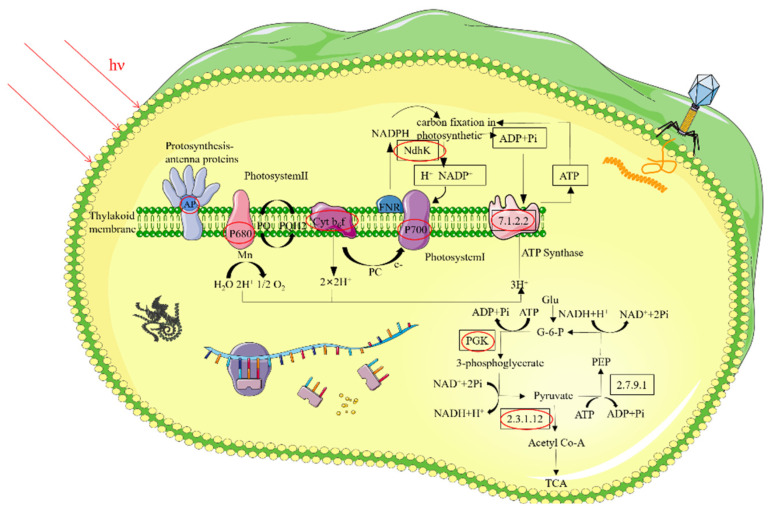
Schematic diagram of key proteins and pathways involved in the lysis of cyanobacteria FACHB-596 cells caused by YongM infestation.

The present study systematically analyzed for the first-time host FACHB-596 DEPs, and their involvement in metabolic pathways, during YongM infestation. This was done at the protein level, using a non-standard quantitative proteomics approach (Figure 6). One hour after infection, expression of cyanobacterial proteins with light-collecting activity was down-regulated, photosynthesis was inhibited and resistance of the host to the exogenous genome was reduced. This may create the ideal conditions for YongM infection and gene replication. The decrease in the expression of ribosomal large subunit proteins L25 and L2 reduced the binding of L2 protein to 23S rRNA and inhibited its peptidyl transferase activity. The host ribosome function was impaired. After 8 h of infestation, the expression of phycocyanin-associated rod-linked proteins and large subunit proteins associated with ribosomal rRNA core stability were upregulated, facilitating light energy uptake and the stable translation of mRNA and sustained protein synthesis. In addition, the expression of S5 protein, closely related to the function of 30S small subunit assembly and able to reduce the rate of translation errors, was also up regulated. This may be consistent with the high vital activity of the cyanophage after 8 h, when it was in the ascending stage of lysis. YongM may induce the ageing of the host in addition to initiate dual lytic enzyme cleavage for rapid passive cleavage and death of the host. Indeed, we found that the expression of the senescence-related protein-cytochrome complex PetA was up-regulated after 8 h of infection. In the carbon fixation pathway, the expression of all DEPs was down regulated. The expression of phosphoglycerate kinase and pyruvate phosphate dikinase was reduced, resulting in an insufficient energy supply for carbon fixation in the host, blocking organic matter synthesis and affecting vital activities. In addition, abnormal expression of the host’s proton transport-related proteins and catalytic enzymes in the oxidative phosphorylation, the glycolysis/gluconeogenesis pathway, and the interconversion of ATP and ADP prevented the host’s metabolic pathways from proceeding normally. 

In conclusion, these results provide systematic information on protein profiling during the invasion and killing of host cyanobacteria by cyanophages. The identification of significant DEPs and cyanophage infestation, cleavage-related effector enzymes in the host cyanobacteria after infestation may provide insights into the design and manipulation of artificial phages against water blooms, as well as genetic resources and stimulate theoretical studies on phage-host interactions.

## Figures and Tables

**Table 1:** Host range analysis of YongM against 33 cyanobacterial strains.

Orders	Family	Species	Strains	Susceptibility	Origin
*Chroococcales*	*Microcystaceae*	*Microcystis aeruginosa*	FACHB-905	+	China
			FACHB-925	−	Australia
			FACHB-942	−	China
			FACHB-469	+	France
			FACHB-924	−	Australia
			FACHB-1326	−	China
			FACHB-912	−	China
		*M. wesenbergii*	FACHB-908	+	China
			FACHB-1112	−	China
			FACHB-1317	−	China
			FACHB-1318	−	China
			FACHB-929	+	Japan
		*M. viridis*	FACHB-979	+	Japan
		*M. Flos-aquae*	FACHB-1028	+	China
		*Microcystis* sp.	FACHB-915	+	France
		*M. elabens*	FACHB-916	−	Japan
		*M. Panniformis*	FACHB-1757	−	China
		*M. ichthyoblabe Kutz*	FACHB-1294	−	China
			FACHB-1409	+	China
	*Chroococcacaea*	*Chroococcus* sp.	FACHB-193	+	China
*Nostocales*	*Aphanizomenonaceae*	*Aphanizomenon flos-aquae*	FACHB-1039	−	China
			FACHB-1040	+	China
		*Anabaena flos-aquae*	FACHB-245	+	USA
		*Anabaena* sp.	FACHB-418	+	France
		*Dolichospermum flos-aquae*	FACHB-1255	+	China
	*Nostocaceae*	*Nostoc* sp.	FACHB-596	+	China
*Oscillatoriale*	*Microcoleaceae*	*Planktothrix agardhii*	FACHB-1166	−	China
			FACHB-920	+	Japan
		*Planktothricoides raciborskii*	FACHB-881	+	China
	*Oscillatoriaceae*	*Oscillatoria planctonica*	FACHB-708	+	China
*Hormogonales*	*Scytonemataceae*	*Plectonema*	FACHB-402	−	USA
			FACHB-240	−	USA
*Synechococcales*	*Synechococcaceae*	*Synechococcus* sp.	FACHB-805	+	Australia

Note: “+” suspective “−” unsuspective.

**Table 2:** The three most significant DEPs in host cyanobacteria induced by YongM infection of FACHB-596 cells. ↑: significantly up-regulated; ↓: significantly down-regulated.

Accession	Protein Name	*p* Value	Adj. *p*-Value	T1:C1	T8:C8
Q8YXF2	ATP-dependent zinc metalloproteinase FtsH	0.008218777	0.52091175	1.5848247↑	
Q8YPZ8	All4042 protein	0.020302562	0.55631214	1.4674468↑	
A0A6P0S0D5	30S ribosomal protein S14	0.014660408	0.53405772	1.4211440↑	
Q8YT39	Alr2887 protein	0.005383913	0.45763262	0.7740167↓	
Q8YTI1	All2736 protein	0.001327486	0.16925451	0.7467759↓	
A0A1Z4KLJ5	50Sribosomal protein L23	0.01222975	0.52091175	0.3643173↓	
A0A1Z4M5R2	Photosystem I P700 chlorophyll-a apolipoprotein A1	0.000000967	0.0000825		3.42825728↑
Q8YYI1	Carbon Dioxide Concentration Mechanism protein	0.000305335	0.00390828		2.98863046↑
Q8YTI1	All2736 protein	0.000000887	0.0000825		2.26210112↑
A0A1Z4IAM0	Unknown functional protein	0.000000195	0.0000499		0.33966838↓
A0A1Z4KN79	Argininosuccinate synthase	0.030938777	0.07920326		0.28915421↓
Q8YWR3	Peroxidase 2 family of proteins/glutaminase	0.009777517	0.04120970		0.25880121↓

**Table 3:** List of DEPs involved in metabolic pathways after infection of host cyanobacteria FACHB-596 cells by YongM.

KEGG Pathway	Protein Name	Protein Description	Upward/Downward
Photosynthesis-antennal proteins	ApcB	Allophycocyanin β subunit	Down
	CpcB	Phycocyanin associated rod junction protein	Up
Ribosomal	L23	Ribosomal large subunit protein L23	Down
	L2	Ribosomal large subunit protein L2	Down
	S14	Ribosomal small subunit protein S14	Up
	L35	Ribosomal large subunit protein L35	Up
	L3	Ribosomal large subunit protein L3	Down
	L4	Ribosomal large subunit protein L4	Down
	L22	Ribosomal large subunit protein L22	Down
	L29	Ribosomal large subunit protein L29	Up
	L5	Ribosomal large subunit protein L5	Down
	S5	Ribosomal small subunit protein S5	Up
	L13	Ribosomal large subunit protein L13	Up
	L1	Ribosomal large subunit protein L1	Up
	L35	Ribosomal large subunit protein L35	Up
	S6	Ribosomal small subunit protein S6	Down
	L21	Ribosomal large subunit protein L21	Up
	S16	Ribosomal small subunit protein S16	Up
	S1	Ribosomal small subunit protein S1	Up
Oxidative phosphorylation	NdhK	NAD (P) H-quinone oxidoreductase subunit K(EC:7.1.1.2)	Down
	alpha	F-type H+/Na+ transporter ATPase subunit α (EC:7.1.2.2 7.2.2.1)	Up
	b	ATPase B subunit	Up
	OSCP	F type H~+ transport ATPase subunit	Up
Photosynthesis	Psb-A	Photosystem II P680 reaction center D1 protein (EC:1.10.3.9)	Down
	Psb-O	Photosystem II oxygen evolution enhancer protein 1	Down
	Psb-U	Photosystem IIPsbU protein	Down
	Psb-V	Photosystem II cytochrome C550	Down
	Psb27	Photosystem IIPsb27 protein	Up
	PsaA	Photosystem I P700 chlorophyll-a apolipoprotein A1	Up
	PetA	Acocytochrome F	Up
	PetE	plastocyanin	Down
	alpha	F-type H+/Na+ transporter ATPase subunit α (EC:7.1.2.2 7.2.2.1)	Up
	b	ATPase B subunit	Up
Carbon fixation	2.7.9.1	Pyruvate, orthophosphate dikinase	Down
	4.1.1.39	Ribulose bisphosphate carboxylase large chain	Down
	2.7.2.3	phosphoglyceric kinase	Down
	2.7.9.1	Pyruvate, orthophosphate dikinase	Down
Glycolysis and gluconeogenesis	2.7.2.3	phosphoglyceric kinase	Down
	2.3.1.12	Pyruvate dehydrogenase E2 component (dihydrolipamidoacetyltransferase)	Up

**Table 4:** Protein profile identification results of YongM.

No.	Location	Description	Score	Coverage
1	20651..21748	putative major capsid protein (Nostoc phage A1)	10,458	86%
2	23869..25389	tail sheath protein (Nostoc phage A1)	4372	46%
3	57421..58554	tail collar protein (Nostoc phage A1)	2265	24%
4	25487..25996	tail tube protein (Nostoc phage A1)	2101	91%
5	18162..19766	hypothetical protein (Nostoc phage A1)	1811	62%
6	29345..32047	lysozyme (Anabaena phage Elbi)	1151	30%
7	5775..7658	alkaline phosphatase D family protein (Salinivenus lutea)	1132	35%
8	26714..28780	tail protein (Nostoc phage A1)	1119	34%
9	60572..61747	baseplate J tail protein (Nostoc phage N1)	1048	44%
10	5256..5762	Capsid fiber protein	1037	61%
11	58577..59965	tail fiber protein (Nostoc phage N1)	995	42%
12	21893..22378	hypothetical protein (Nostoc phage A1)	925	49%
13	19898..20644	putative outer membrane protein (Nostoc phage A1)	912	49%
14	61784..62131	lysozyme (Nostoc phage N1)	672	73%
15	4580..5233	hypothetical protein (Nostoc phage A1)	453	24%
16	32813..33661	hypothetical protein (Nostoc phage N1)	376	37%
17	8358..9086	hypothetical protein (Nostoc phage A1)	288	20%
18	59997..60572	tail collar protein (Nostoc phage A1)	198	30%
19	23110..23832	hypothetical protein (Nostoc phage A1)	169	25%
20	7738..8361	hypothetical protein (Nostoc phage A1)	156	40%
21	62202..62531	hypothetical protein	101	64%
22	32074..32541	hypothetical protein	100	26%
23	63660..64472	Baseplate structural protein	71	11%
24	22375..22701	putative tail-component	66	19%
25	22706..23113	hypothetical protein	57	18%
26	15042..15680	hypothetical protein (Nostoc phage A1)	29	6%
27	42619..43221	hypothetical protein (Microcystis phage Me-ZS1)	29	6%
28	55094..57100	DNA polymerase delta catalytic subunit	19	1%
29	3..722	Thymidylate kinase (EC 2.7.4.9)	19	7%
30	20661..26102	tail length tape-measure protein (Microcystis phage Me-ZS1)	16	2%
31	41382..41699	hypothetical protein (Microcystis phage Me-ZS1)	14	6%
32	26900..27886	tail tube protein (Microcystis phage Me-ZS1)	13	4%

## Data Availability

Not applicable.
